# Factor Xa Mediates Calcium Flux in Endothelial Cells and is Potentiated by Igg From Patients With Lupus and/or Antiphospholipid Syndrome

**DOI:** 10.1038/s41598-017-11315-9

**Published:** 2017-09-07

**Authors:** Bahar Artim-Esen, Natalia Smoktunowicz, Thomas McDonnell, Vera M. Ripoll, Charis Pericleous, Ian Mackie, Eifion Robinson, David Isenberg, Anisur Rahman, Yiannis Ioannou, Rachel C. Chambers, Ian Giles

**Affiliations:** 1 Center for Rheumatology Research, Rayne Institute, University College London, London, UK; 2 Division of Rheumatology, Department of Internal Medicine, Istanbul Faculty of Medicine, Istanbul University, Istanbul, Turkey; 3 Center for Inflammation and Tissue Repair, Rayne Institute, University College London, London, UK; 4 Imperial College London, Kingston upon Thames, United Kingdom; 5 Haemostasis Research Unit, Department of Haematology, University College London, London, UK; 6 Department of Chemistry, Faculty of Maths and Physical Sciences, University College London, London, UK

## Abstract

Factor (F) Xa reactive IgG isolated from patients with antiphospholipid syndrome (APS) display higher avidity binding to FXa with greater coagulant effects compared to systemic lupus erythematosus (SLE) non APS IgG. FXa signalling via activation of protease-activated receptors (PAR) leads to increased intracellular calcium (Ca^2+^). Therefore, we measured alterations in Ca^2+^ levels in human umbilical vein endothelial cells (HUVEC) following FXa-mediated PAR activation and investigated whether FXa reactive IgG from patients with APS or SLE/APS- alter these responses. We observed concentration-dependent induction of Ca^2+^ release by FXa that was potentiated by APS-IgG and SLE/APS- IgG compared to healthy control subjects’ IgG, and FXa alone. APS-IgG and SLE/APS- IgG increased FXa mediated NFκB signalling and this effect was fully-retained in the affinity purified anti-FXa IgG sub-fraction. Antagonism of PAR-1 and PAR-2 reduced FXa-induced Ca^2+^ release. Treatment with a specific FXa inhibitor, hydroxychloroquine or fluvastatin significantly reduced FXa-induced and IgG-potentiated Ca^2+^ release. In conclusion, PAR-1 and PAR-2 are involved in FXa-mediated intracellular Ca^2+^ release in HUVEC and FXa reactive IgG from patients with APS and/or SLE potentiate this effect. Further work is required to explore the potential use of IgG FXa reactivity as a novel biomarker to stratify treatment with FXa inhibitors in these patients.

## Introduction

Pathogenic antiphospholipid antibodies (aPL) interact with various cells including monocytes, endothelial cells (EC) and trophoblast cells leading to the recruitment of cell surface receptors and activation of intracellular signalling pathways^1^. These interactions give rise to the major clinical manifestations of vascular thrombosis and/or pregnancy morbidity that define the antiphospholipid syndrome (APS). Evidence has demonstrated the importance of inflammation in the pathogenesis of the APS through activation of complement and a family of G-protein coupled receptors, protease-activated receptors (PARs)^2, 3^, four of which (PAR-1-4) have been identified^4^. PARs are activated by serine proteinase (SP) enzymes involved in haemostasis^5^ and inflammation^4^; including thrombin, activated Factor (F) VIIa, FIXa, FXa and FXIIa.

Increased expression of PARs (particularly PAR-2) has been reported in APS monocytes^6^ and tissue factor (TF)/FVIIa/PAR-2 mediated signalling in neutrophils has been shown to be important in the pathogenesis of pregnancy morbidity in a murine model of APS^3^. Furthermore, a panel of monoclonal human aPL displayed cross-reactivity with SP, including thrombin, FIXa and FXa^7–11^ and several monoclonal human aPL inhibited the inactivation of procoagulant SP and functional activities of anticoagulant/fibrinolytic SP^8, 9, 12, 13^. We found that amino-acid sequence changes in the antigen binding sites of recombinant human monoclonal aPL which altered the pattern of binding to thrombin predicted pathogenicity in mice^14^. Other studies have identified that between 13–54% of sera from patients with APS bind different SP^9, 12, 15^

Therefore, it has been suggested that some aPL may recognise the catalytic domain of SP leading to dysregulation of haemostasis and vascular thrombosis in APS. Given that cellular responses elicited through activation of PARs by thrombin and FXa influence pathways responsible for inflammation and haemostasis, modulation of PAR activation in the presence of anti-SP antibodies may be important in the pathogenesis of APS. We previously showed that thrombin reactive IgG were significantly elevated in patients with APS and in patients with SLE who were aPL positive but lacked APS (SLE/aPL+) compared to healthy controls. Furthermore, IgG purified from patients with APS displayed higher avidity for thrombin, and significantly inhibited antithrombin-III inactivation of thrombin compared with IgG from SLE/aPL+ (without APS) and healthy control subjects (HC)^16^.

More recently, we have shown that serum from patients with APS and patients with SLE (without APS) had significantly increased IgG reactivity for FXa compared with controls^17^. Polyclonal IgG purified from serum of patients with APS showed higher avidity binding to FXa and greater effects upon the enzymatic and coagulant activity of FXa compared with polyclonal IgG isolated from patients with SLE who lacked APS. In those experiments, however, we did not study the effects of IgG on the actions exerted by FXa on cells via PARs.

Considering the central position of FXa in the coagulation cascade^18^ and that FXa acting via PARs influences inflammation and thrombosis by activating a wide range of cell types including EC^19^, we hypothesized that polyclonal IgG with FXa reactivity may alter these cellular actions in patients with APS and/or SLE. To test this hypothesis, we required an experimental system to measure the effects of FXa and IgG on PAR mediated activation in EC. Therefore, we measured real-time intracellular calcium (Ca^2+^) flux, which is widely used to measure the activation of G protein coupled receptors such as PARs on various cells. First, we fully characterised FXa-PAR mediated alterations in intracellular Ca^2+^ levels in HUVEC in the absence of IgG and then investigated the ability of polyclonal IgG with FXa reactivity isolated from serum of patients with APS or SLE (no APS) to alter these FXa-mediated responses. We then measured the functional effects of this FXa reactive IgG and affinity purified anti-(a)FXa IgG upon FXa-PAR mediated NFκB signalling in HUVEC. Next we examined the effects of a selective FXa inhibitor, antistasin, on the FXa-mediated Ca^2+^ release in HUVEC in the presence or absence of these patient-derived IgG. We also examined the effects of hydroxychloroquine (HCQ) and fluvastatin, since these drugs have been proposed to reduce thrombotic risk in APS but whose mechanism of action is unclear and for statins has been linked with PAR expression in animal models of APS^3^.

## Results

### Clinical and laboratory features of subjects studied

The characteristics of patients and controls are shown in Table 1. Of patients with APS, 7 had primary APS and 7 had SLE/APS; whilst 10 had a history of thrombosis (6 vascular thrombosis only, 2 vascular thrombosis and pregnancy morbidity and 2 catastrophic APS) and 4 of pregnancy morbidity only. Of the 14 patients with SLE and no APS (SLE/APS-), 8 were aPL positive (SLE/aPL+) and 6 aPL negative (SLE/aPL−) with an average disease duration of 19.3 (+/−12.8 SD) years and no APS manifestations. Of the five SLE/APS- patients listed as Lupus Anticoagulant (LA) positive, four were weakly positive once and then negative on repeated testing; only one patient had strong persistent positivity more than 12 weeks apart. Of these 5 patients: none were positive for anti-β2GPI antibodies; three had single LA positivity; one had weak non-persistent aCL positivity; one with strong persistent LA positivity had strong persistent IgM aCL positivity; and none have developed any manifestations of APS despite a disease duration ranging from 11–20 years in these five patients alone. All patients were selected on the basis of their positive binding to FXa. HC (n = 8) were randomly chosen from a group of 40 healthy individuals all of whom had no FXa reactive antibodies. Purified IgG was used for the experiments.

**Table 1:** Clinical and laboratory features of patients and controls studied.

DIAGNOSIS	APS (n = 14)	SLE/APS- (n = 14)	HC (n = 8)
Age, mean years ± SD	45 ± 11	45 ± 13	37 ± 15
Sex, male/female	1/13	2/12	2/6
VT only, n (%)	6 (43)	3(21)*	0
PM only, n (%)	4 (29)	2(14)**	0
VT + PM, n (%)	2 (14)	0	0
CAPS, n (%)	2 (14)	0	0
Other ARD	SLE 7	0	0
Manifestations SLE, (n)	Muc (6), Haem (7), Joint (7), Ser (2), renal (1), CNS (1)	Joint (13), Haem (11), Ser (8), Muc (7), renal (5), CNS (2)	—
aCL, mean GPL units	40 ± 31	13 ± 11	8
Anti-β2GPI, mean AU	26 ± 23	12 ± 9	2.5
LA, n (%)	7 (50)	5 (35)	0
Anti-FXa+, mean activity (%)***	36 ± 18	38 ± 20	0
Anti-Thr+, mean activity (%)***	17 ± 34	22 ± 19	1.7 ± 3

aCL = Anticardiolipin antibody; APS = Antiphospholipid syndrome; ARD = Autoimmune rheumatic disease; ATIII = Antithrombin III; AU = Arbitrary units; β2GPI = Anti- β2-glycoprotein I; CAPS = Catastrophic antiphospholipid syndrome; CNS = central nervous system; FXa = Factor Xa; GPL = IgG phospholipid; Haem = haematological; HC = Healthy control subjects; LA = Lupus anticoagulant; Muc = mucocutaneous; NA = Not available; PM = Pregnancy morbidity; SD = Standard deviation; Ser = serositis; SLE = Systemic lupus erythematosus; VT = Vascular thrombosis; *SLE/APS- patients with VT were all aPL negative and did not satisfy APS classification criteria; **Non-APS miscarriages; ***expressed as percentage binding of a positive control.

### APS IgG reduces FXa activity

Our previous findings of the effects of APS-IgG on FXa *in vitro* ^17^ are summarised in Table 2. APS IgG significantly reduced FXa activity (P < 0.0001 for APS versus HC, and P = 0.0008 for APS versus SLE IgG) and antithrombin-III mediated inhibition of FXa activity (P < 0.0001 for APS versus SLE and APS versus HC IgG) and significantly prolonged the clotting time compared to HC IgG (p < 0.0001) and SLE/APS- IgG (p = 0.04).

**Table 2:** Summary of effects of IgG upon FXa activity *in vitro*.

	Inhibition of FXa activity (mean ± SEM; %)	Inhibition of FXa activity in the presence of ATIII* (mean ± SEM; %)	FXa activity –clotting time (seconds)
APS-IgG	9.7 ± 0.89	62.03 ± 1.36	74.2 ± 4.4
SLE-IgG	7.07 ± 1.28	81.40 ± 0.32	63.6 ± 2.7
HC-IgG	2.58 ± 0.6	80.16 ± 1.09	26.8 ± 0.6

APS: Antiphospholipid syndrome, AT-III: Antithrombin III, HC: Healthy control, SEM: Standard error of mean,

SLE: Systemic lupus erythematosus.

*All compared to FXa-only (without IgG) inhibition by AT-III.

Of the 14 IgG preparations in each APS and SLE/APS- group, 4 in APS and 7 in SLE/APS- displayed weak thrombin binding of less than 20% above the threshold for positivity compared with FXa binding which was between 40 to 60% above the relative cut-off for positivity (data not shown).

### FXa-induced Ca^2+^ release in HUVEC is attenuated by PAR-1 and PAR-2 antagonists

**Figure 1:**
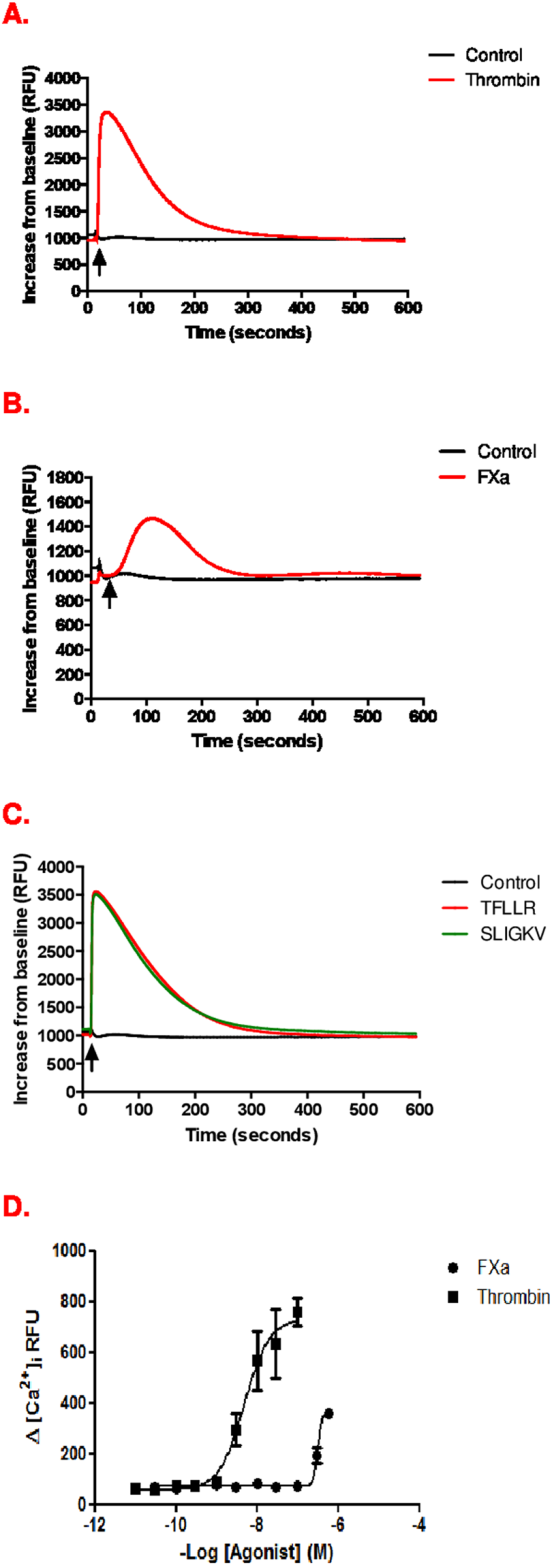
HUVEC response to FXa, thrombin and PAR agonist peptides. Fluo-4 –loaded HUVEC were stimulated by PAR-1 and PAR-2 agonists and fluorescence intensity readings recorded continuously for 10 minutes as described. Data are shown as representative traces of intracellular Ca^2+^ release upon stimulation with (**A**) Thrombin (10 nM); (**B**) FXa (150 nM) (**C**) PAR-1 agonist peptide TFLLR and PAR-2 agonist peptide SLIGKV. (**D**) Concentration-response curves in HUVECs following Thrombin (3–30 nM) and FXa (30–150 nM) stimulation.

**Figure 2:**
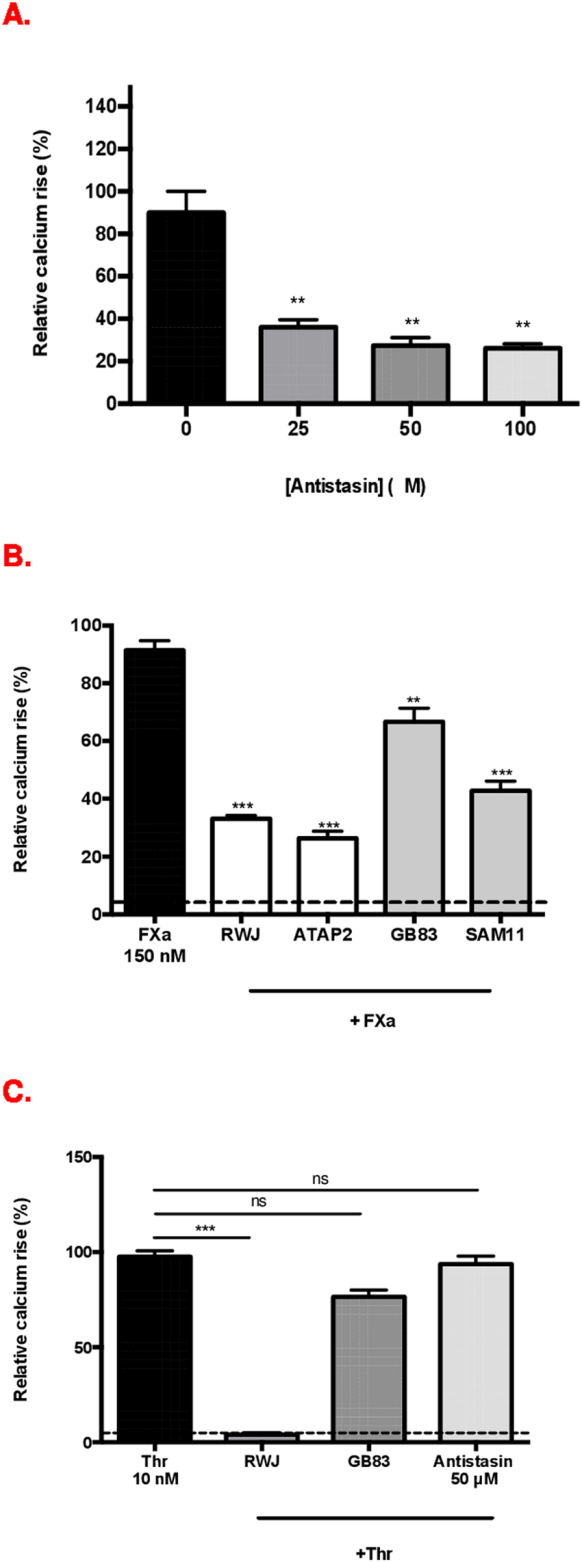
(**A**) FXa-mediated calcium responses are inhibited by specific FXa inhibitor Antistasin related peptide at concentrations 25, 50 and 100 μM. (**B**) Inhibition of FXa-induced Ca^2+^ release by PAR inhibitors and blocking antibodies. (**C**) Inhibition of thrombin-induced Ca^2+^ release by PAR inhibitors and Antistasin. Data are plotted with mean ± standard error of mean (SEM), 1-way ANOVA **p < 0.05, ***p < 0.001 when compared to FXa alone at all concentrations compared to FXa or thrombin alone. Dotted line represents control buffer. ATAP2: PAR-1 blocking antibody; FXa: Factor Xa; GB83: PAR-2 selective antagonist; RWJ–58259: PAR-1 antagonist; SAM11: PAR-2 blocking antibody, ns: non-significant.

It was important to first fully characterise the kinetics of FXa-PAR-mediated Ca^2+^ release and the relative contribution of PAR-1 and PAR-2 activation in HUVEC. The kinetics of FXa-promoted intracellular Ca^2+^ secretion in HUVEC were different from those of thrombin and PAR-1 and PAR-2 agonist peptide (AP), with a lower magnitude, longer lag time and longer duration of action (Fig. 1A to D). Incubation of FXa with Antistasin, a potent, selective FXa proteolytic inhibitor caused significant concentration-dependent inhibition of of Ca^2+^ release: 59% at 25 μM; 69% at 50 μM; and 70% at 100 μM Antistasin (p < 0.05 at all concentrations compared to FXa alone) (Fig. 2A). Antistasin alone did not induce Ca^2+^ release. Inhibition with the direct thrombin inhibitor Hirudin, did not cause a change in the effect of 150 nm FXa on Ca^2+^ flux, thus confirming that the results were independent of thrombin (data not shown). Furthermore, our experiments with 4 different chromogenic substrates to detect FXa and thrombin activity in the FXa preparations used showed that FXa had the expected reactivity with substrates having greater relative sensitivity and specificity for FXa and no activity on substrates with high thrombin sensitivity. All FXa activity was blocked by a high affinity, very specific FXa inhibitor, rivaroxaban, while there was no effect of the thrombin inhibitor hirudin (Supplementary data Fig. 1A,B and C). Taken together, these data allowed us to conclude that FXa-induces Ca^2+^ release at high concentrations and that this is dependent on FXa proteolytic activity.

We next examined the potential contribution of either PAR-1 or PAR-2 to the FXa-mediated Ca^2+^ release using pharmacological antagonists (RWJ-58259 for PAR-1 and GB83 for PAR-2) and antibodies (ATAP2 for PAR-1 and SAM11 for PAR-2). The strongest reduction in FXa-induced Ca^2+^ release was observed with PAR-1 inhibition (~60%). PAR-2 inhibition with SAM11 and GB83 also significantly reduced this response (Fig. 2B). In contrast, thrombin-induced Ca^2+^ release was strongly inhibited by PAR-1 antagonism of RWJ-58259 (p < 0.001) but no significant reduction was observed with the PAR-2 antagonist, GB83 (Fig. 2C). These studies revealed that FXa-mediated Ca2+ release is both PAR-1 and PAR-2 dependent and confirm that Antistasin specifically inhibits FXa.

### APS-IgG potentiates FXa-mediated intracellular Ca^2+^ signalling

**Figure 3:**
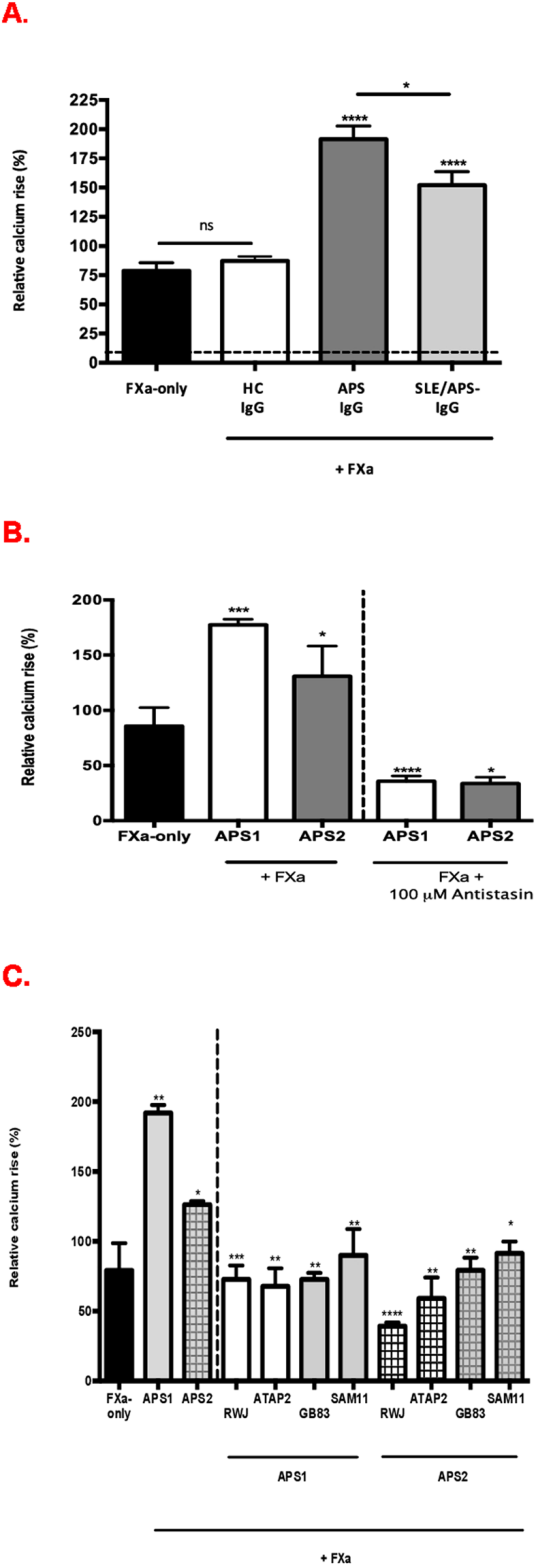
(**A**) IgG (used at 200 μg/ml) effect on FXa (150 nM)-induced Ca^2+^ release. ****p < 0.0001 when APS-IgG (*191.5* ± *11.3%)* compared to FXa-only control (*78.7* ± *6.9%*) and to HC IgG (*87.1* ± *3.7%)*, *p = 0.02 when compared to SLE IgG (*152.1* ± *11.5%)*, dotted line represents IgG alone. Each bar represents mean ± standard error of mean (SEM) of n = 14 APS IgG, n = 14 SLE IgG and n = 8 HC IgG (**B**) Antistasin inhibited IgG potentiated FXa-calcium mediated responses. IgG that potently inhibited Ca^2+^ release were selected (shown as APS1 and APS2). Comparison of calcium release: by APS1 with inhibited vs unhibited FXa: ****p < 0.0001, by APS2 with inhibited vs uninhibited FXa: *p = 0.03. Potentiation of FXa-mediated release by IgG is also significant: APS1 ***p = 0.0008, APS2 *p = 0.03. Plotted with mean ± SEM (**C**) Inhibition of IgG potentiated FXa-induced Ca^2+^ release by PAR inhibitors and blocking antibodies. IgG that potently inhibited Ca^2+^ release were selected (shown as APS1 and APS2). ***p = 0.0006 APS1+RWJ-58259 vs APS1, **p < 0.05 APS1 + GB83/PAR 1 BL AB/ PAR2 BL AB vs APS1; ****p < 0.0001 APS2+RWJ-58259 vs APS2, **p < 0.05 APS1 + GB83/PAR 1 BL AB, *p = 0.01 APS2 + PAR2 BL AB vs APS2; Potentiation of FXa-mediated release by IgG is significant: APS1 **p = 0.005, APS2 *p = 0.04. Plotted with mean ± standard error of mean. APS: Antiphospholipid syndrome; ATAP2: PAR-1 blocking antibody; FXa: Factor Xa; GB83: PAR-2 selective antagonist; HC: Healthy control; **ns:** non-significant; RWJ–58259: PAR-1 antagonist; SAM11: PAR-2 blocking antibody; SLE: Systemic lupus erythematosus.

The effects of polyclonal IgG isolated from patients with APS (n = 14) and FXa reactive antibodies and from patients with SLE/APS- (n = 14) and FXa reactive antibodies upon the FXa-PAR interaction were then determined. APS IgG significantly potentiated FXa-induced Ca^2+^ release compared to SLE/APS- IgG (p = 0.02), HC IgG (p < 0.001) and FXa-only stimulation (p < 0.001). Significant potentiation of FXa-induced Ca^2+^ release was also observed with SLE/APS- IgG compared to FXa alone and to HC IgG (p < 0.001 for both) (Fig. 3A). No Ca^2+^ release was observed by the effect of purified IgG on HUVEC in the absence of FXa. To exclude an LA mediated effect between the IgG groups we confirmed that SLE/APS-/LA− vs SLE/APS-/LA+ and APS/LA+ vs APS/LA- IgG did not display any significant differences (158.2 +/− 17.28 vs 141.1 +/− 9.4 and 242.4 +/− 57 vs 203 +/− 34 respectively). Comparison of intracellular Ca^2+^ release induction by HC IgG to FXa-only stimulation did not reveal any significant difference (Fig. 3A). IgG alone did not have an effect upon Ca flux (Fig. 3A).

We then investigated whether the effects of selected IgG (shown as APS1 and APS2) that potently enhanced FXa-mediated Ca^2+^ release were reduced in the presence of the FXa inhibitor. There was a significant reduction of IgG-FXa-mediated Ca^2+^ release in the presence of Antistasin compared to IgG-FXa alone from: 177.4 ± 3.5% to 35.74 ± 2.7% (p < 0.0001) for APS1; and 130.8 ± 19.3% to 33.6 ± 4.1% (p = 0.03) for APS2 (Fig. 3B). This APS IgG potentiated FXa-induced Ca^2+^ release was also significantly reduced in the presence of PAR-1 and PAR-2 antagonists and antibodies (Fig. 3C).

### Hydroxychloroquine and fluvastatin inhibit IgG potentiated FXa-mediated Ca^2+^ signalling

HCQ and statins have been proposed as potential anti-thrombotic therapies in APS, although the cellular mechanisms underlying their anti-thrombotic effects are only partially characterised and for statins have been linked with PAR expression in animal models of APS^3^. Therefore, we examined whether these drugs may interfere with FXa-PAR induced Ca^2+^ release in HUVEC.

**Figure 4:**
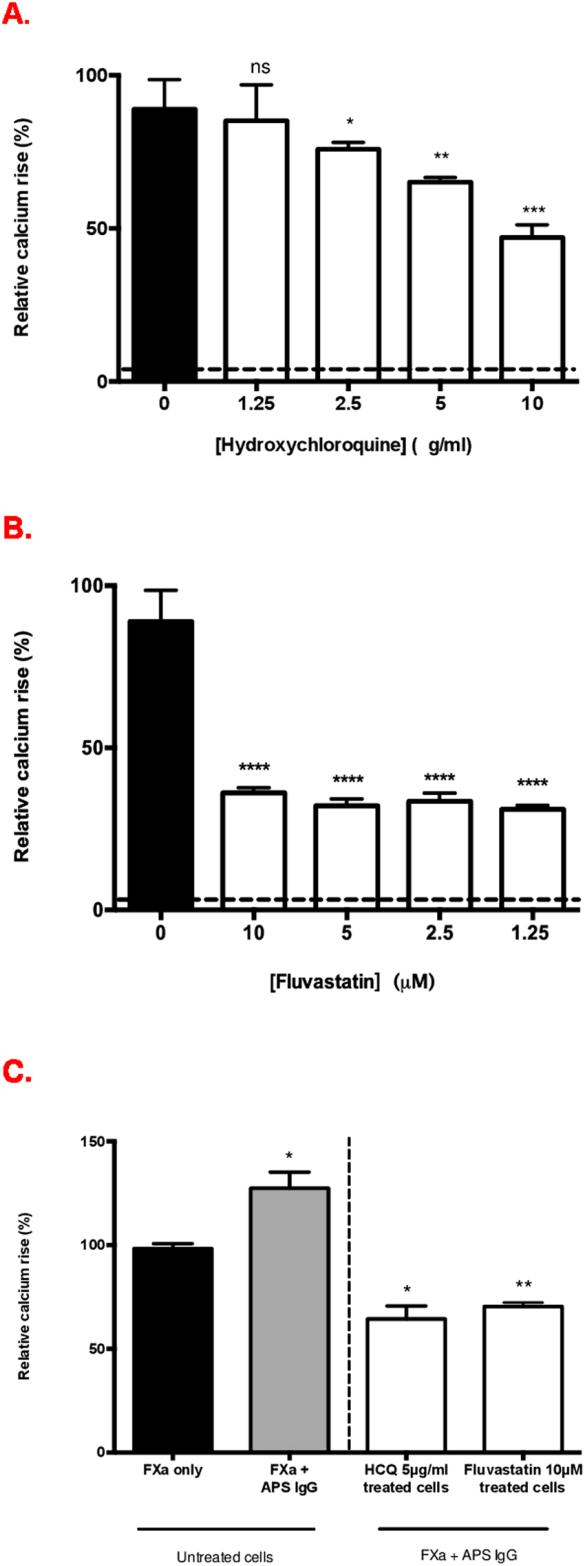
Effect of drugs on FXa (150 nM)-mediated Ca^2+^ signaling. Cells were treated with HCQ and fluvastatin for 20 hours and stimulated with FXa 150 nM (**A**) Hydroxychloroquine (HCQ) (used at 1.25, 2.5, 5, 10 μg/ml) *p = 0.02, **p = 0.001, ***p < 0.001 for comparison of FXa stimulation of untreated cells vs 2.5, 5, 10 μg/ml HCQ treated HUVEC respectively. (**B**) Fluvastatin (used at 1.25, 2.5, 5, 10 μM) ****p < 0.0001 for comparison of FXa stimulation of untreated cells vs 2.5, 5, 10 μM fluvastatin treated HUVEC respectively. (**C**) Inhibition of IgG-potentiated intracellular Ca^2+^ release in HCQ 5 μg/ml and fluvastatin 10 μM treated cells. IgG that mediated the strongest effect on Ca^2+^ release was selected. FXa + APS IgG vs FXa-only stimulation in untreated cells, *p = 0.03; FXa + APS IgG in untreated cells vs HCQ treated cells, *p = 0.01, vs fluvastatin treated cells **p = 0.009. Plotted with mean ± standard error of mean. Dotted line represents drug alone in graphs A and B.

First, we pre-incubated HUVEC with varying concentrations of HCQ for 20 hours and then untreated and treated cells were stimulated with FXa alone. Comparison of FXa stimulation of untreated HUVEC to HCQ-treated HUVEC (Fig. 4A) revealed a significant concentration-dependent inhibition of intracellular Ca^2+^ mobilisation from 2.5 μg/ml onwards.

In a separate set of experiments, we pre-incubated cells with different concentrations of fluvastatin for 20 hours and then untreated and fluvastatin-exposed cells were stimulated with FXa alone. Fluvastatin significantly reduced FXa-induced Ca^2+^ release (p < 0.0001) compared with FXa stimulation of untreated cells (Fig. 4B) at all concentrations tested. Cell proliferation assay confirmed viability of cells in the presence of both drugs (Supplementary data Fig. 2A and B)

To determine the effect of these drugs on IgG potentiation of FXa-mediated Ca^2+^ release, the drug treated cells were exposed to selected APS IgG (4 samples) that induced the highest Ca^2+^ release with FXa (Fig. 4C). IgG potentiation of FXa induced intracellular Ca^2+^ release was significantly reduced by: 63% with HCQ (p = 0.01); and 57% with Fluvastatin (p = 0.009).

### Affinity purified anti-FXa IgG potentiates FXa-mediated intracellular signalling

**Figure 5:**
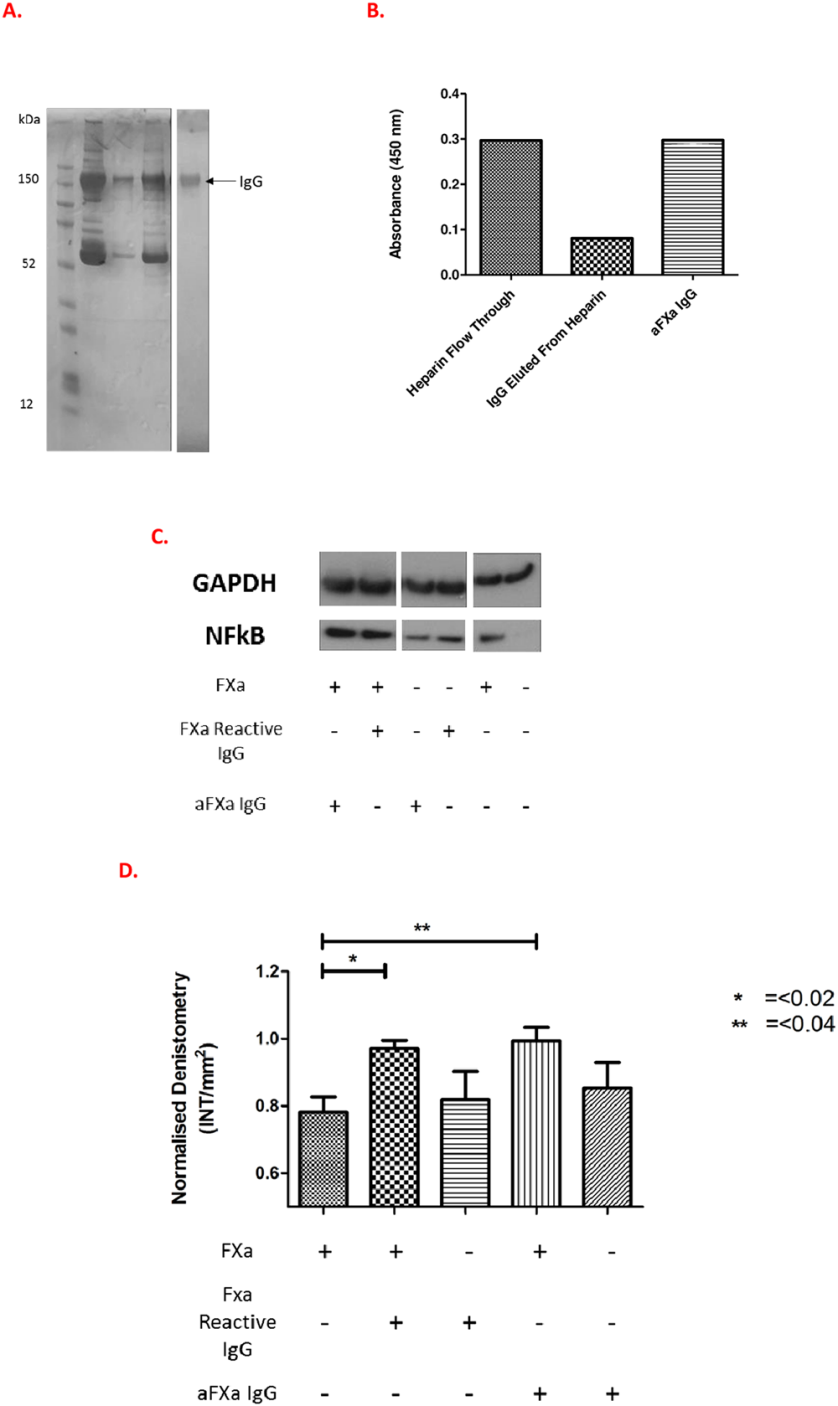
Affinity purification of anti-FXa IgG and effects on FXa-mediated intracellular signaling. The anti-(a)FXa sub-fraction was affinity purified from n = 3 patients with the hightest FXa binding. (**A**) Gel showing flowthrough of serum passed through the Heparin column (lane 2), wash-step (lane 3), purification through aFXa column (lane 4) and subsequent IgG purification by Sepharose protein G (lane 5). (**B**) A graph showing the aFXa activity of IgG fractions: column 1, flow-through of heparin column; column 2, fraction containing IgG non-specifically bound to heparin; and column 3, affinity purified aFXa IgG. The values are OD at 450 nm. (**C**) Representative (n = 1) western blot and (**D**) densitometric analysis (n = 3) showing that FXa reactive whole IgG and affinity purified aFXa IgG significantly (p < 0.05) upregulates phosphorylation of NFkB in comparison to FXa alone or IgG alone.

To confirm that the IgG has FXa-PAR mediated effects that are specific to the anti-FXa antibody sub-fraction, anti-(a)FXa IgG were afinity purified from n = 3 patients with the highest levels of FXa binding, including both APS and SLE/APS- patients. These samples were randomly selected and a limited number tested due to the low yield of IgG from this process and ethical restrictions on volume of serum collection. Following purification (Fig. 5A) and elution of aFXa IgG we confirmed its binding to FXa (Fig. 5B).

We then measured the functional effects of IgG upon FXa-PAR mediated NFκB signalling in HUVEC as previous studies have shown the relevance of this pathway in FXa mediated signalling in EC (Jiang *et al*., J Surg res 2011;169:319 & Bukowska *et al*., Molecular and Cellular Pharmacology 2013;718:114). We found that aFXa IgG upregulated NFκB phosphorylation in the presence of FXa and this effect was fully retained by the affinity purified aFXa IgG specific sub-fraction (Fig. 5C and D).

## Discussion

In this study we have characterised FXa-PAR-mediated effects on intracellular Ca^2+^ signalling in HUVEC and examined the effects of polyclonal IgG from FXa reactive antibody positive patients with SLE and/or APS, as well as HCQ and fluvastatin on this response. We have shown that FXa stimulation of HUVEC is mediated via PAR-1 and PAR-2 dependent signalling and that this response is enhanced by IgG from FXa reactive antibody positive patients with APS as well as SLE/APS- and can be blocked by a specific FXa proteolytic inhibitor, antistasin, HCQ and fluvastatin. Furthermore, we developed a method to purify the specific aFXa sub-fraction of these antibodies, demonstrated that they fully-retain binding to FXa and have FXa dependent functional effects upon FXa-PAR mediated signalling in EC.

Previously, we identified that IgG isolated from FXa reactive antibody positive patients with APS have differential avidity and effects upon the enzymatic and coagulant activity of FXa compared with IgG isolated from patients with SLE who lacked APS. Given that FXa exerts PAR-mediated cellular effects upon EC and these cells are important in the pathogenesis of the APS, we studied the effects of FXa reactive IgG upon FXa activation of intracellular Ca^2+^ signalling responses in EC. PAR activation requires proteolytic cleavage by extracellular proteinases^20^ to mediate cellular responses. Cleavage of the receptor leads to the intra-molecular binding of a tethered ligand and signalling via coupling to G-proteins, and the mobilisation of intracellular Ca^2+^ ^21, 22^. EC functions are highly dependent on Ca^2+^ ^21^ and even minor changes in intracellular Ca^2+^ can trigger pathological events ranging from inflammatory responses to cell death^23–26^.

Thrombin signalling is well characterised and mediated through PAR-1, 3 and 4^21^. EC express high levels of PAR-1 and PAR-2 and the prototypic PAR-1 agonist is thrombin whereas PAR-2 can be activated by a number of other proteinases. FXa has been demonstrated to act on PARs in multiple different vascular cell types^27, 28^. It has been shown to activate PAR-2^29, 30^ and PAR-1 on EC and induce Ca^2+^ responses^29, 31, 32^. Furthermore, FXa elicits protective signalling responses in EC directly via PAR-2 and indirectly via endothelial protein C receptor (EPCR) dependent recruitment of PAR-1^33^. Another interesting feature is that different activators of the same receptor do not induce identical cellular responses^26, 34, 35^. A possible explanation for these different cellular responses was provided by studies demonstrating that different agonists of PAR1 selectively activate different downstream G-protein pathways by their ability to alter receptor/G protein affinity within the same receptor in a process known as functional selectivity^36^.

Therefore, it was initially important to characterise the relative contribution of PAR-1 and PAR-2 to FXa-mediated signalling in EC before proceeding to experiments with IgG from patients with FXa reactive antibody positive patients.

To achieve this aim, we used agents to specifically inhibit PARs. Two different types of approaches were used: monoclonal antibodies which block cleavage of the tethered ligand and thus activation of PAR-1 (ATAP2)^37^ and PAR-2 (SAM11)^38^; and small molecule antagonist, RWJ-58259, a competitive and reversible PAR-1 antagonist that blocks the interaction between the PAR-1 tethered ligand and the second extracellular loop of PAR-1^39^. For PAR-2, we used the reversible antagonist, GB83^40^. We found that inhibition of PAR-1 and PAR-2 receptors by corresponding antagonists and cleavage blocking antibodies caused a significant reduction in FXa-mediated Ca^2+^ release in HUVEC. These findings implicate both PAR-1 and PAR-2 in FXa-mediated signalling.

Having characterised the PAR receptor system involved in FXa signalling in HUVEC, we then examined the effects of IgG on this response. We showed that the FXa stimulation of HUVEC in the presence of IgG isolated from FXa reactive antibody positive patients with APS caused a significant increase in intracellular Ca^2+^ release compared with SLE/APS- and HC IgG. We believe that these IgG mediated effects may be explained by their binding to FXa rather than any cross-reactivity with thrombin for several reasons. First, only 11 of the 28 APS and SLE/APS- IgG tested displayed binding to thrombin (which was weak in all 11 cases) in contrast to their strong FXa binding seen in all 28 cases. Second, the strongest effect on FXa-mediated Ca^2+^ release was observed with APS IgG, which included only four samples that bound thrombin. Potentiation of Ca^2+^ release was observed with all samples tested whether or not they bound thrombin. Thirdly, this effect was likely to be FXa-mediated because it was blocked by Antistasin, a small, disulphide cross-linked protein of 119 amino acid residues, which selectively and potently inhibits FXa^41^. The absence of inhibition by a direct thrombin inhibitor and the lack of thrombin activity (using amidolytic substrates) in the FXa preparations also confirm the FXa dependency of the effect. Lastly, comparison of LA- IgG to LA+IgG revealed a similar enhancement of FXa-mediated Ca^2+^ release, thus confirming that we did not merely observe a LA effect.

To further study whether these IgG mediated effects were FXa specific we developed a method to affinity purify aFXa IgG by passage through a heparin (to remove IgG that binds non-specifically to the negatively charged resin) and then FXa column to isolate the aFXa sub-fraction (Fig. 5A). The (n = 3) aFXa IgG tested, fully-retained FXa binding (Fig. 5B) and the ability to upregulate FXa mediated NFκB signalling in EC compared with the FXa reactive IgG from which it was purified (Fig. 5C and D). We examined these signalling pathways because previously studies have shown them to be important in FXa-PAR mediated activation of endothelilal cells and human atrial tissue^42, 43^. The effects of aFXa IgG upon these pathways are directly relevant to our findings with FXa reactive IgG upon FXa-mediated Ca^2+^ release as they represent downstream signalling pathways of FXa-PAR activation in EC, thus further evidence that the IgG effects are mediated through binding to FXa.

The yield of IgG from affinity purification is substantially reduced compared to that of whole IgG and does not allow for extensive testing in biological assays. For instance, our previous experience with affinity purification of the anti-domain (aD)I sub-fraction of IgG the final yield of aDI IgG is appreciably less (140 mcg/ml) compared with whole IgG (10–12 mg/ml) so much larger volumes of serum are required to produce the final amount of affinity purified IgG for testing of one sample at similar concentrations to whole IgG in functional assays^44^. In our aFXa affinity purification method we obtain similar reductions in yield with final concentrations of aFXa IgG at 100 mcg/ml. Due to ethical limitations upon the volume of blood collection we were only able to produce limited amounts of aFXa IgG for testing in a reduced number of experiments.

The reduced yield obtained with affinity purification of IgG may explain why most other mechanistic studies of IgG mediated disease in SLE/APS utilise whole IgG fractions. Of the few studies that carry out affinity purification it is usually to test a limited number of samples in validation experiments as we have done. For instance, in addition to our own study of n = 1 aDI^44^ other authors studied effects of: n = 3 affinity purified anti-β2GPI IgG onf MyD88 signalling in EC^45^ n = 6 affinity purified aCL on platelet glycoprotein expression^46^; and n = 2 affinity purified anti-β2GPI antibodies upon *in vitro* LA activity^47^. Therefore, we have utilised a well-established methodology to study the biological effect of a larger cohort of whole IgG and then confirm the specificity ifs effect using a smaller number of affinity purified IgG.

Previously, we have shown that the effects of IgG from patients with primary APS and those with SLE associated APS on monocyte signalling pathways and the proteome were similar^48, 49^. Likewise, we did not find a significant difference in the effects of IgG from primary APS and SLE/APS- on FXa-mediated Ca^2+^ release (data not shown). Our finding that SLE/APS- IgG also significantly potentiated FXa-mediated Ca^2+^ release compared to HC IgG may indicate that these IgG are also important in SLE. SLE is characterized by an increased risk of cardiovascular disease, which is not fully explained by traditional risk factors therefore consideration of immunological factors is important^50^. Given that PAR activation may contribute to the pathogenesis of cardiovascular disease^51^.and the central importance of FXa in mediating inflammation and thrombosis via PAR activation, it is tempting to speculate that anti-FXa positive IgG may be important in the pathogenesis of cardiovascular disease in SLE.

A direct FXa inhibitor (rivaroxaban) is now widely used as primary and secondary thrombo-prophylaxis in several clinical settings. A recent trial has suggested that it may be safely used in the management of patients with thrombotic APS and might offer a convenient alternative to warfarin in this subgroup of patients with APS^52^.Therefore, our findings of the effect of FXa inhibitors upon *in-vitro* cellular effects of FXa-reactive APS IgG may provide additional evidence to support the use of FXa inhibitors in patients with APS.

In addition, we demonstrated that the FXa-mediated effects of APS-IgG upon EC were inhibited by statins and HCQ. The use of these drugs has been proposed in APS to ameliorate the hemorrhagic risk associated with anticoagulant drugs. In particular, statins are not only potent inhibitors of cholesterol synthesis but they have also been shown to modify the function of EC and platelets by decreasing the expression of adhesion molecules, inhibiting TF expression and down-regulating inflammatory cytokines after treatment with aPL^53^. Simvastatin and pravastatin have been shown to decrease TF and PAR-2 expression on neutrophils and prevent pregnancy loss in mice^3^. Similarly, HCQ has been shown to reduce the extent of thrombosis in an animal model of injury-induced thrombosis in APS, reverse aPL-induced platelet activation and to protect the EC annexin A5 anticoagulant shield from disruption by aPL^54^. Our new findings provide evidence that these drugs may additionally inhibit the biological effect of APS-IgG through their inhibitory effects on FXa-induced Ca^2+^ release. Interestingly, HCQ has been shown to alter Ca^2+^ signalling in T cells^55^ and macrophages^56^ and both of these cells express and respond to PAR activation^57^.

The FXa-mediated potentiation of Ca^2+^ release in HUVEC by FXa-reactive APS IgG implicates PAR-1 and PAR-2 in this endothelial response in the context of APS. To our knowledge there are no published reports of APS-related PAR expression in EC. Increased PAR-2 expression has been shown in monocytes isolated from patients with thrombotic APS compared with non-thrombotic APS, thrombosis without APS and healthy controls^6^. This same study also demonstrated a correlation between the levels of PAR-2 expression, aCL IgG titers and TF expression in these patients and found that APS-IgG significantly increased expression of PAR-1 and PAR-2 on healthy monocytes. Inhibition of PAR-2 prevented the aCL-induced expression of TF^6^. In addition, PAR-2 activation in the TF/FVIIa/PAR-2 complex on neutrophils has been shown to increase neutrophil activation, trophoblast injury and fetal death in aPL treated mice^3^. Soluble FXa^58^ and FXa engaged in the ternary TF/VIIa/FXa complex^59^ activate both PAR-1 and PAR-2 leading to cellular effects that are important in modulating inflammation, cell survival/proliferation, fibrosis and angiogenesis as well as thrombosis^58^. PAR activation, may therefore, have direct relevance to the pathogenesis of the APS.

The concentration of FXa required to induce intracellular Ca^2+^ release may appear to be supraphysiologic. However, cofactors and biological surfaces exert significant effects on the activity of coagulation factors^60^. In accordance with this FXa signalling is dependent on cell type and cofactor expression^61^. Its signalling is mediated via both PAR1 and PAR2 in endothelial cells but high exogenous concentrations are required for receptor activation as it is a relatively inefficient activator of PAR1^29, 61, 62^. Additionally, under *in vivo* conditions, FXa, when complexed to TF and FVIIa is five times more potent at activating PAR1 compared to FXa alone which is thought to be related to more efficient recruitment of FXa to the cell membrane^62^. Furthermore, limited data exist on the local concentrations of coagulation factors during pathological states but it is possible that much higher concentrations are required at sites of cellular damage and activation.

Although the number of samples in our study may be considered small they are comparable to those of other studies that have isolated IgG from patients to examine their biologic effects upon target cells. For instance, Cuadrado *et al*., compared the effects of n = 7 APS IgG with an IgG sample pooled from n = 10 healthy controls upon cultured monocytes^63^; whilst Meroni *et al*., studied the effects of n = 3 APS-IgG and a healthy control IgG on HUVEC signalling mechanisms^64^. In our systematic review of 29 studies critically analysing the strength of the evidence that specific receptors and signalling pathways are important in APS pathogenesis, it was striking that APS-IgG samples were obtained from very small numbers (usually five or less) of individual patients^65^. Therefore, the ideal of testing IgG from larger numbers of patients and matched controls remains very difficult for ourselves and others to achieve.

In summary we have characterised FXa-PAR mediated activation of EC and found IgG with FXa reactivity from patients with APS and SLE/APS to alter it in a PAR-mediated manner. Furthermore, IgG effects are blocked by a specific FXa inhibitor as well as HCQ and fluvastatin. Future work is now required to explore whether FXa reactivity may allow stratified FXa inhibitory therapy in those patients and further dissect the anti-FXa specificity of the effect.

## Materials and Methods

### Patients and healthy controls

Serum was isolated from selected patients attending University College London Hospital with APS, (n = 14) and with SLE and no APS (SLE/APS-), (n = 14) from a larger cohort selected on the basis of their positive binding to FXa, and 8 healthy control subjects (HC) who had no FXa reactivity. All patients satisfied relevant disease classification criteria – APS^66^ and SLE^67^. Informed consent and full ethical approval from the local ethics board were obtained (National Research Ethics Committee- London Hampstead, reference number 12/LO/0373). All methods were performed in accordance with the relevant guidelines and regulations.

### Immunologic characterisation and purification of IgG

Coagulation factors were from Haematologic Technologies, USA, unless otherwise stated. Porcine gelatin, bovine serum albumin (BSA) and conjugated antibodies were from Sigma-Aldrich, UK. Chromogenic substrates for ELISA were from KPL, USA.

IgG was protein G purified (Pierce, UK), dialysed in phosphate-buffered saline (PBS) and the concentration determined by spectrophotometry. Further purification of aFXa IgG was performed by passage of IgG though a heparin column to remove IgG that binds non-specifically to the negatively charged resin, followed by purification of the remaining IgG fraction though an immobilised FXa column and subsequent dialysis to PBS. The presence of IgG directed against cardiolipin^14^, β2-glycoprotein I (β2-GPI)^14^ thrombin^16^ and FXa was measured by ELISA as previously described^17^. The cut-off of positivity for all ELISAs was determined from a cohort of forty HC. All samples were tested in duplicate and considered positive when the test optical density (OD) minus the background OD exceeded the mean OD + 3 standard deviations (SD) of HC. Results were expressed as percentage binding of a positive control.

### Clotting and functional assay for FXa activity

Effects of FXa-reactive IgG on FXa-activated clotting time (ACT) was measured as described previously^17^. The effects of anti-FXa reactive IgG on FXa activity were studied by digestion of a chromogenic substrate S-2765 (Chromogenix; DiaPharma) both in the absence and presence of ATIII and the degree of colour change used to quantify the activity of FXa^17^.

### Human umbilical vein endothelial cells, tissue culture

HUVEC were purchased from Lonza, USA and were seeded into 75 cm^2^ tissue culture flasks (Costar, Cambridge, MA) in EBM-2 basal media (Lonza, USA) containing 10% fetal calf serum, L- glutamine, standard antibiotics and endothelial cell growth supplement, EGM-2 (Lonza, USA). For calcium signaling experiments, HUVEC of up to passage 4 were trypsinized, seeded at a density of 10^4^ cells/well (200 μL/well) in 96-well flat-bottom plates in air containing 5% CO_2_ at 37 °C and were grown to 75% confluency for 48 hours. Cells were serum-starved the night before the experiment and subsequently stimulated with FXa under a range of different conditions, as described below.

### Measurement of intracellular calcium levels

Intracellular Ca^2+^ levels were assessed using the Fluo-4 AM kit (Invitrogen, UK) as described previously^68^. Briefly, the Ca^2+^ -binding dye was re-suspended in assay buffer (20 mM HEPES in HBSS) supplemented with 2.5 mM probenecid. Cells were loaded with the Ca^2+^ sensitive dye and incubated for 30 minutes at 37 °C and 30 minutes at RT. PAR-1 and PAR-2- mediated changes in intracellular Ca^2+^ were monitored using a fluorescent image plate reader (FLIPRTetra, Molecular Devices (UK) Limited, Wokingham, UK) following stimulation with 150 nM FXa, 10 nM α-thrombin, 100 μM PAR-1 agonist peptide (AP) (TFLLR) or 100 μM PAR-2 AP (SLIGKV) (AP both from Bachem AG, Switzerland)^69^. The concentrations of α-thrombin and PAR-AP were selected based on our previous experience with these agonists in this experimental system in EC. The dose of FXa was determined by optimisation experiments.

### Experimental conditions

FXa was incubated for 45 minutes with the IgG (200 μg/ml) from patients and controls prior to HUVEC stimulation and changes in intracellular Ca^2+^ release were monitored for 10 minutes following stimulation and compared to stimulation with FXa alone. This concentration of IgG was selected because it was consistent with the dose used in our previous FXa study^17^ and is well within the (200–500 μg/ml) range used by ourselves and others studying the effects of APS-IgG upon cultured HUVEC^70, 71^.

The effects of IgG were also measured in the presence of PAR or FXa antagonists or HCQ or fluvastatin. PAR-1 cleavage-blocking monoclonal antibody (ATAP2)^37^ and PAR-2 cleavage blocking antibody (SAM11)^38^ (Santa Cruz Biotechnology, USA) were both used at 10 μg/ml and the selective human PAR-1 antagonist, RWJ – 58259 (synthesized in-house by the UCL Department of Chemistry)^39^ was used at 3 µM and a selective PAR-2 antagonist, GB83 (Axon Medchem, USA)^40^ was used at 50 µM. These reagents were added to the cells during the loading period, one hour before the experiment. FXa was preincubated with the specific FXa inhibitor Antistasin-Related Peptide (D-Arg32) (from Bachem, Germany) at 25, 50 or 100 μM for one hour before it was added to the cells to determine the contribution of FXa proteolytic activity. HCQ (used at 1.25, 2.5, 5, 10 μg/ml) and fluvastatin (used at 0.1, 0.3, 1, 3 μM; from Sigma-Aldrich, UK), were pre-incubated with cells for 20 hours. Experiments were performed at least in triplicate for each condition and data plotted as relative fluorescence units expressed in % compared to Ca^2+^ mobilization in FXa-only stimulated HUVEC. Results are representative of at least three independent experiments.

### Measurement of intracellular signalling

The effects of aFXa IgG upon FXa mediated EC signalling pathways were tested by incubating aFXa IgG (150 µg/ml) with or without FXa (40 nM) for 30 minutes before stimulating HUVEC in 10% FCS-EGM media. Cell lysates were obtained at 20 and 40 minutes to measure phosphorylated & total forms of NFkB.

### Statistical analysis of data

Data are presented as means ± SEM and were analysed in GraphPad Prism using ANOVA (multiple group comparisons) followed by Tukey HSD post hoc analysis or Student’s t-test (single group comparisons). Differences between means with a p-value < 0.05 were considered significant.

## Electronic supplementary material

Functional assay for FXa activity and MTS assay protocol

## References

[1] Giannakopoulos B, Passam F, Rahgozar S, Krilis SA (2007). Current concepts on the pathogenesis of the antiphospholipid syndrome. Blood..

[2] Redecha P (2007). Tissue factor: a link between C5a and neutrophil activation in antiphospholipid antibody induced fetal injury. Blood..

[3] Redecha P, Franzke CW, Ruf W, Mackman N, Girardi G (2008). Neutrophil activation by the tissue factor/Factor VIIa/PAR2 axis mediates fetal death in a mouse model of antiphospholipid syndrome. J Clin Invest..

[4] Ma L, Dorling A (2012). The roles of thrombin and protease-activated receptors in inflammation. Semin Immunopathol..

[5] Walsh PN, Ahmad SS (2002). Proteases in blood clotting. Essays Biochem..

[6] Lopez-Pedrera C (2010). Differential expression of protease-activated receptors in monocytes from patients with primary antiphospholipid syndrome. Arthritis Rheum..

[7] Lu CS (2005). Identification of polyclonal and monoclonal antibodies against tissue plasminogen activator in the antiphospholipid syndrome. Arthritis Rheum..

[8] Lin WS (2007). Some antiphospholipid antibodies recognize conformational epitopes shared by beta2-glycoprotein I and the homologous catalytic domains of several serine proteases. Arthritis Rheum..

[9] Hwang KK (2001). Identification of anti-thrombin antibodies in the antiphospholipid syndrome that interfere with the inactivation of thrombin by antithrombin. J Immunol..

[10] Yang YH (2009). Novel autoantibodies against the activated coagulation factor IX (FIXa) in the antiphospholipid syndrome that interpose the FIXa regulation by antithrombin. J Immunol..

[11] Yang YH (2006). Antibodies against the activated coagulation factor X (FXa) in the antiphospholipid syndrome that interfere with the FXa inactivation by antithrombin. J Immunol..

[12] Yang CD (2004). Identification of anti-plasmin antibodies in the antiphospholipid syndrome that inhibit degradation of fibrin. J Immunol..

[13] Hwang KK (2003). A thrombin-cross-reactive anticardiolipin antibody binds to and inhibits the anticoagulant function of activated protein C. Arthritis Rheum..

[14] Giles I (2009). Thrombin Binding Predicts the Effects of Sequence Changes in a Human Monoclonal Antiphospholipid Antibody on Its *In Vivo* Biologic Actions. J Immunol..

[15] Cugno M (2004). Antibodies to tissue-type plasminogen activator (tPA) in patients with antiphospholipid syndrome: evidence of interaction between the antibodies and the catalytic domain of tPA in 2 patients. Blood..

[16] Lambrianides A (2011). Interactions of Human Monoclonal and Polyclonal Antiphospholipid Antibodies With Serine Proteases Involved in Hemostasis. Arthritis Rheum..

[17] Artim-Esen B (2015). Anti-FXa antibodies in patients with antiphospholipid syndrome and their effects on coagulation assays. Arthritis Res Ther..

[18] Borensztajn K, Peppelenbosch MP, Spek CA (2008). Factor Xa: at the crossroads between coagulation and signaling in physiology and disease. Trends Mol Med..

[19] Krupiczojc MA, Scotton CJ, Chambers RC (2008). Coagulation signalling following tissue injury: focus on the role of factor Xa. Int J Biochem Cell Biol..

[20] Bauer KA (1989). Detection of factor X activation in humans. Blood..

[21] Soh UJK, Dores MR, Chen B, Trejo J (2010). Signal transduction by protease-activated receptors. Br J Pharmacol..

[22] Berridge MJ, Lipp P, Bootman MD (2000). The versatility and universality of calcium signaling. Nat Rev Mol Cell Biol..

[23] Tran QK, Ohashi K, Watanabe H (2000). Calcium signaling in endothelial cells. Cardiovasc Res..

[24] Deng X, Mercer PF, Scotton CJ, Gilchrist A, Chambers RC (2008). Thrombin induces fibroblast CCL2/JE production and release via coupling of PAR1 to Galphaq and cooperation between ERK1/2 and Rho kinase signaling pathways. Mol Biol Cell..

[25] Bootman MD (2001). Calcium signalling-an overview. Semin Cell Dev Biol..

[26] Blanc-Brude OP (2005). Factor Xa stimulates fibroblast procollagen production, proliferation, and calcium signaling via PAR1 activation. Exp cell res..

[27] Rana S, Yang L, Hassanian SM, Rezaie AE (2012). Determinants of the specificity of protease-activated receptors 1 and 2 signaling by factor Xa and thrombin. J Cell Biochem..

[28] Manithody C, Yang L, Rezaie AE (2012). Identification of exosite residues of factor Xa involved in recognition of PAR-2 on endothelial cells. Biocemistry..

[29] Camerer E, Kataoka H, Kahn M, Lease K, Coughlin SR (2002). Genetic evidence that protease-activated receptors mediate factor Xa signaling in endothelial cells. J Biol Chem..

[30] Kawabata A (2001). Factor Xa-evoked relaxation in rat aorta: involvement of PAR-2. Biochem. Biophys. Res. Commun..

[31] Senden NHM (1998). FXa induces cytokine production and expression of adhesion molecules by human umblical vein endothelial cells. J Immunol.

[32] McLean K, Schirm S, Johns A, Morser J, Light DR (2001). FXa-induced responses in vascular Wall cells are PAR-mediated and inhibited by ZK-807834. Thromb Res..

[33] Bae JS, Yang L, Rezaie AR (2010). Factor X/Xa elicits protective signaling responses in endothelial cells directly via PAR-2 and indirectly via endothelial protein C receptor-dependent recruitment of PAR-1. J Biol Chem..

[34] Riewald M, Ruf W (2005). Protease-activated receptor-1 signaling by activated protein C in cytokine-perturbed endothelial cells is distinct from thrombin signaling. J Biol Chem..

[35] Daubi V (2006). Factor Xa and thrombin evoke additive calcium and proinflammatory responses in endothelial cells subjected to coagulation. Biochem Biophys Acta..

[36] McLaughlin JN (2005). Functional selectivity of G protein signaling by agonist peptides and thrombin for the protease-activated receptor-1. J Biol Chem..

[37] O’Brien PJ (2000). Thrombin responses in human endothelial cells. Contributions from receptors other than PAR1 include the transactivation of PAR2 by thrombin-cleaved PAR1. J Biol Chem..

[38] Adams MN, Pagel CN, Mackie EJ, Hooper JD (2012). Evaluation of antibodies directed against human protease-activated receptor-2. Naunyn Schmiedebergs Arch Pharmacol..

[39] Damiano BP, Derian CK, Maryanoff BE, Zhang HC, Gordon PA (2003). RWJ-58259: a selective antagonist of protease activated receptor-1. Cardiovasc Drug Rev..

[40] Barry GD (2010). Novel agonists and antagonists for human protease activated receptor 2. J Med Chem..

[41] Ohta N, Brush M, Jacobs JW (1994). Interaction of antistasin-related peptides with FXa: identification of a core inhibitory sequence. Thromb Haemost..

[42] Jiang R (2011). Factor Xa induces tissue factor expression in endothelial cells by P44/42 MAPK and NF-κB-dependent pathways. J Surg res.

[43] Bukowska A (2013). Coagulation factor Xa induces an inflammatory signalling by activation of protease-activated receptors in human atrial tissue. Eur J Pharmacol.

[44] Pericleous C (2015). Proof-of-concept study demonstrating the pathogenicity of affinity-purified IgG antibodies directed to domain I of β2-glycoprotein I in a mouse model of anti-phospholipid antibody-induced thrombosis. Rheumatology.

[45] Raschi E (2003). Role of the MyD88 transduction signaling pathway in endothelial activation by antiphospholipid antibodies. Blood.

[46] Espinola RG, Pierangeli SS, Gharavi AE, Harris EN (2002). Hydroxychloroquine reverses platelet activation induced by human IgG antiphospholipid antibodies. Thromb Haem.

[47] Viveros ME, Cabiedes J, Reyes E, Cabral AR (2005). Activated protein C resistance and lupus anticoagulant activity induced by plasma and purified monospecific human IgG anti-beta2-glycoprotein-I antibodies. Rev Invest Clin.

[48] Lambrianides A (2010). Effects of polyclonal IgG derived from patients with different clinical types of the antiphospholipid syndrome on monocyte signaling pathways. J Immunol..

[49] Ripoll VM (2014). Changes in regulation of human monocyte proteins in response to IgG from patients with antiphospholipid syndrome. Blood..

[50] Narshi CB, Giles IP, Rahman A (2011). The endothelium: an interface between autoimmunity and atherosclerosis in systemic lupus erythematosus?. Lupus..

[51] Leger AJ, Covic L, Kuliopulos A (2006). Protease-activated receptors in cardiovascular diseases. Circulation..

[52] Cohen H (2016). **RAPS** trial investigators. Rivaroxaban versus warfarin to treat patients with antiphospholipid syndrome, with or without systemic lupus erythematosus (RAPS): a randomized, controlled, open-label, phase 2–3, non-inferiority trial. Lancet Haematol..

[53] Lopez-Pedrera C, Ruiz-Limon P, Aguirre MA, Rodriguez-Ariza A, Cuadrado MJ (2012). Potential use of statins in the treatment of antiphospholipid syndrome. Curr Rheumatol Rep..

[54] Rand JH (2010). Hydroxychloroquine protects the annexin A5 anticoagulant shield from disruption by antiphospholipid antibodies: evidence for a novel effect for an old antimalarial drug. Blood..

[55] Goldman FD (2000). Hydroxychloroquine inhibits calcium signals in T cells: a new mechanism to explain its immunomodulatory properties. Blood..

[56] Misra UK, Gawdi G, Pizzo SV (1997). Chloroquine, quinine and quinidine inhibit calcium release from macrophage intracellular stores by blocking inositol 1,4,5-trisphosphate binding to its receptor. J Cell Biochem..

[57] Shpacovitch V, Feld M, Hollenberg MD, Luger TA, Steinhoff M (2008). Role of protease-activated receptors in inflammatory responses, innate and adaptive immunity. J Leukoc Biol..

[58] Sevigny LM (2011). Protease-activated receptor-2 modulates protease-activatedreceptor-1-driven neointimal hyperplasia. Arterioscler Thromb Vasc Biol..

[59] Riewald M, Ruf W (2001). Mechanistic coupling of protease signaling and initiation of coagulation by tissue factor. Proc Natl Acad Sci USA.

[60] Umme A (2010). Molecular Intercommunication between the Complement and Coagulation Systems. J Immunol..

[61] Riewald M, Ruf W (2002). Orchestration of coagulation protease signaling by tissue factor. Trends Cardiovasc Med..

[62] Riewald M, Ruf W (2001). Mechanistic coupling of protease signaling and initiation of coagulation by tissue factor. Proc Natl Acad Sci USA.

[63] Cuadrado MJ (2006). Vascular endothelial growth factor expression in monocytes from patients with primary antiphospholipid syndrome. J Thromb Haemost..

[64] Raschi E (2003). Role of the MyD88 transduction signaling pathway in endothelial activation by antiphospholipid antibodies. Blood..

[65] Poulton K, Rahman A, Giles I (2012). Examining how antiphospholipid antibodies activate intracellular signaling pathways: a systematic review. Semin Arth Rheum..

[66] Miyakis S (2006). International consensus statement on an update of the classification criteria for definite antiphospholipid syndrome (APS). J Thromb Haemost..

[67] Hochberg MC (1997). Updating the American College of Rheumatology revised criteria for the classification of systemic lupus erythematosus. Arthritis Rheum..

[68] Ortiz-Stern A, Deng X, Smoktunowicz N, Mercer PF, Chambers RC (2012). PAR-1-dependent and PAR-independent pro-inflammatory signaling in human lung fibroblasts exposed to thrombin. J Cell Physio..

[69] Hollenberg MD, Saifeddine M, al-Ani B, Kawabata A (1997). Proteinase-activated receptors: structural requirements for activity, receptor cross-reactivity, and receptor selectivity of receptor-activating peptides. Can J Physiol Pharmacol..

[70] Vega-Ostertag ME (2007). Role of p38 mitogen-activated protein kinase in antiphospholipid antibody-mediated thrombosis and endothelial cell activation. J Thromb Haemost..

[71] Pericleous C (2013). Endothelial microparticle release is stimulated *in vitro* by purified IgG from patients with the antiphospholipid syndrome. Thromb Haemost..

