# Supplementary material for: Factor Xa Mediates Calcium Flux in Endothelial Cells and is Potentiated by Igg From Patients With Lupus and/or Antiphospholipid Syndrome
Source: Sci Rep. 2017 Sep 7;7:10788. doi: 10.1038/s41598-017-11315-9 (PMC5589732; doi:10.1038/s41598-017-11315-9)

FACTOR Xa MEDIATES CALCIUM FLUX IN ENDOTHELIAL CELLS AND IS POTENTIATED BY IgG FROM PATIENTS WITH LUPUS AND/OR ANTIPHOSPHOLIPID SYNDROME

AUTHORS

Bahar ARTIM-ESEN* [1]Research fellow at Center for Rheumatology Research, Rayne Institute, University College London, London, UK

[2] MD, Division of Rheumatology, Department of Internal Medicine, Istanbul Faculty of Medicine, Istanbul University, Istanbul, Turkey

e-mail: bahar.artimesen@istanbul.edu.tr

Natalia SMOKTUNOWICZ PhD, Center for Inflammation and Tissue Repair, Rayne Institute, University College London, London, UK

e-mail: Natalia_smoktunowicz@yahoo.com

Thomas McDONNELL PhD, Center for Rheumatology Research, Rayne Institute, University College London, London, UK

e-mail: thomas.mcdonnell.11@ucl.ac.uk

Vera M RIPOLL PhD, Center for Rheumatology Research, Rayne Institute, University College London, London, UK

e-mail: v.ripoll-nunez@ucl.ac.uk

Charis PERICLEOUS PhD, Center for Rheumatology Research, Rayne Institute, University College London, London, UK

The current affiliation for Charis Pericleous is Imperial College London, Kingston upon Thames, United Kingdom

e-mail: c.pericleous@imperial.ac.uk

Ian MACKIE PhD, Haemostasis Research Unit, Department of Haematology, University College London, UK

e-mail: i.mackie@ucl.ac.uk

Eifion ROBINSON PhD, Department of Chemistry, Faculty of Maths and Physical Sciences, University College London, UK
e-mail: eifion.robinson.09@ucl.ac.uk

David ISENBERG MD, FRCP, Center for Rheumatology Research, Rayne Institute, University College London, London, UK

d.isenberg@ucl.ac.uk

Anisur RAHMAN PhD, FRCP, Center for Rheumatology Research, Rayne Institute, University College London, London, UK

Anisur.rahman@ucl.ac.uk

Yiannis IOANNOU PhD, FRCP, Arthritis Research UK Centre for Adolescent Rheumatology, University College London (UCL), UCL Hospital and Great Ormond Street Hospital, London, UK

e-mail: y.ioannou@ucl.ac.uk

Rachel C CHAMBERS PhD, Center for Inflammation and Tissue Repair, Rayne Institute, University College London, London, UK

r.chambers@ucl.ac.uk

Ian GILES PhD, MRCP, Center for Rheumatology Research, Rayne Institute, University College London, London, UK

e-mail: i.giles@ucl.ac.uk

RUNNING TITLE: **FXa-MEDIATED CELLULAR EFFECTS IN SLE AND/OR APS**

Author of Correspondance *

Bahar Artim-Esen

E-mail: bahar.artimesen@istanbul.edu.tr **Tel:** 0090-212 414 23 00

Address:

Istanbul University, Istanbul Faculty of Medicine, Department of Internal Medicine, Division of Rheumatology 34098 Çapa, Fatih / Istanbul-TURKEY

Supplementary methods

Functional assay for FXa activity

The chromogenic substrates S-2337, S-2765(Chromogenix; Quadratech Diagnostics Ltd, Epsom, UK), PefafluorXa (Pentapharm; Quadratech Diagnostics Ltd) and S-2238 ((Chromogenix; Quadratech Diagnostics Ltd, Epsom, UK) were used to detect FXa and thrombin activity in the FXa preparations. Briefly, human FXa was diluted in 50mM Tris, 130mM NaCl, pH 8.3, containing 0.5% bovine serum albumin (Sigma, A7030) buffer at RT. Subsequently, the chromogenic substrate was added, and generation of p-nitroaniline was monitored at 405 nm. The activity of FXa was determined based on the rate of hydrolysis of the chromogenic substrate from the linear range of absorbance at 405 nm over time. Rivaroxaban and hirudin were used to block the cleavage of chromogenic substrates. Each chromogenic substrate was used at a final concentration of 1mM, FXa of 50nM, rivaroxaban of 1.1uM, and hirudin of 120uM in the reaction mixture.

MTS assay protocol

Human umbilical vein endothelial cells (HUVEC) cells were treated with hydroxychloroquine (HCQ) and statin, at a range of concentrations 0 – 100 microg/ml and 0 – 10 microM, respectively, for 24 hours. The cell viability was subsequently assessed using CellTiter 96 Aqueous One Solution Cell Proliferation Assay (Promega, USA), according to the manufacturer's protocol. Briefly, 20 microl of the CellTiter 96 Aqueous One Solution Reagent was added to the cells and the absorbance was recorded at 490 nm following 1.5 - hour incubation at 37°C, 5% CO_2.

Supplementary Figures

Supplementary Fig 1 Thrombin and FXa activity in the FXa preparations
Four different chromogenic substrates, namely, S2337, S2238, S2765, and PefafluorXa used to detect thrombin and FXa activity in the FXa preparations showed that FXa had the expected reactivity with substrates having greater relative higher sensitivity and specificity for FXa and that had no activity on the substrate with high thrombin sensitivity **(A)**. Rivaroxaban, the specific FXa inhibitor, blocks the cleavage of FXa sensitive substrates with a greater relative sensitivity and specificity **(B)** but Hirudin, the specific thrombin inhibitor, does not have any effect **(C). S2337, S2765** and **Pefa Xa** to detect FXa activity; **S2238** to detect thrombin activity.

A.

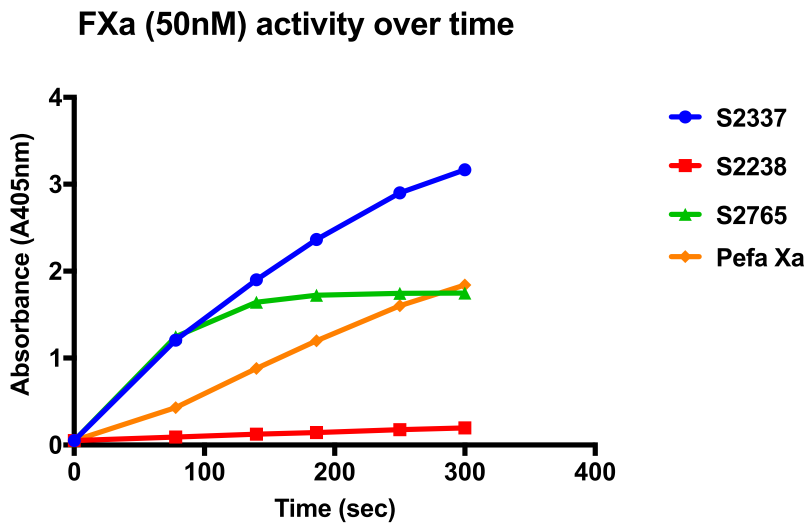

.

B.

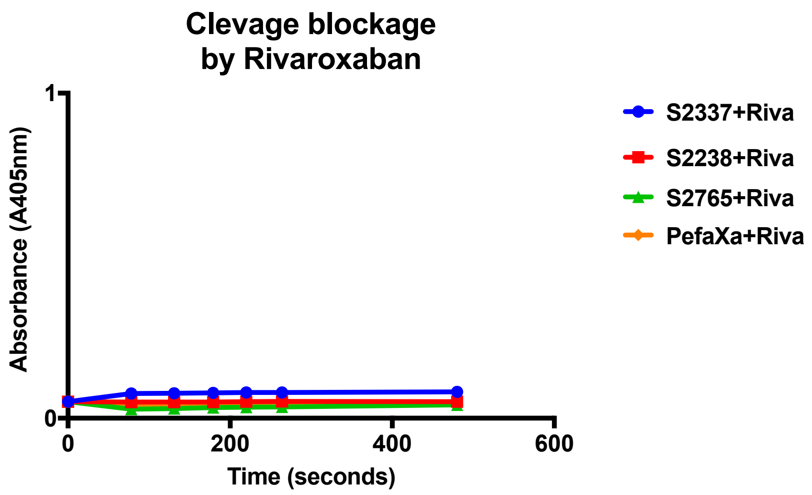

C.

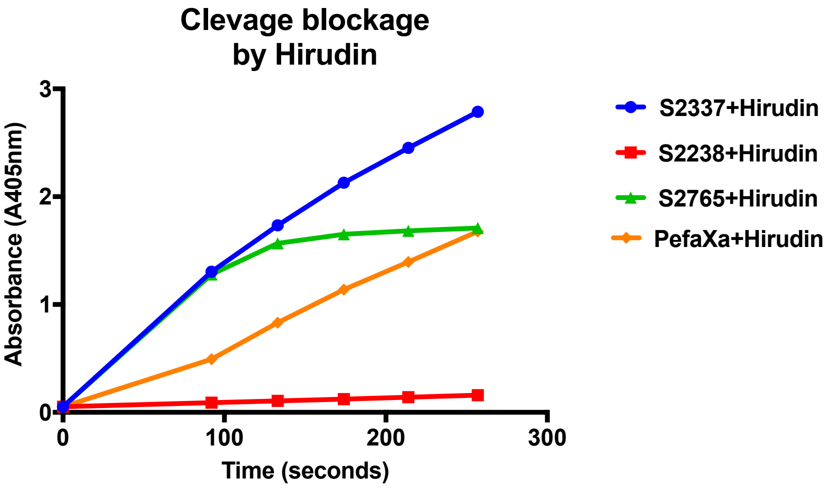
Clevage blockage
by Hirudin
Absorbance (A405nm)
0
1
2
3
0
100
200
300
Time (seconds)
S2337+Hirudin
S2238+Hirudin
S2765+Hirudin
PefaXa+Hirudin

Supplementary Fig 2

HCQ and statin do not affect HUVEC proliferation. MTS assay was performed to assess the effect of HCQ and statin treatment on HUVEC proliferation. HCQ decreased cell proliferation at highest concentrations only **(A),** while statin did not have any significant effect on cell proliferation at any concentrations used **(B).** Data are representative of 4 replicate wells, +/-, 1-way ANOVA,***p<0.001. HCQ: Hydroxychloroquine, HUVEC: Human umbilical vein endothelial cells

A.

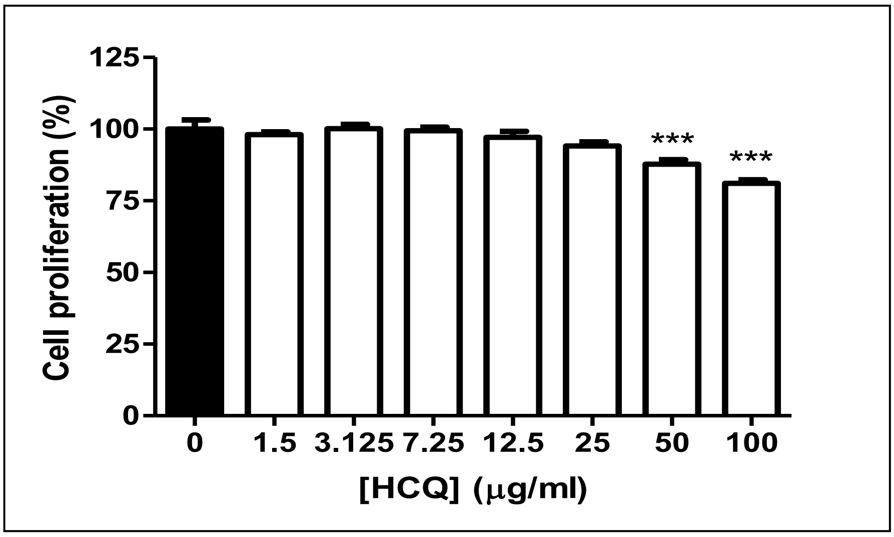

B.

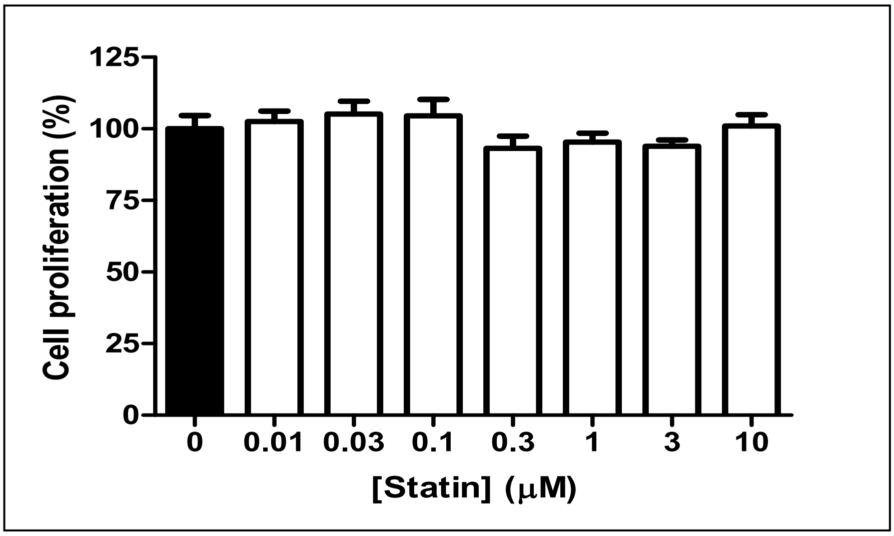

Supplement: Supplementary file 1 — Functional assay for FXa activity and MTS assay protocol [file 41598_2017_11315_MOESM1_ESM.pdf]
